# Effectiveness of *Helicobacter pylori* eradication in the prevention of primary gastric cancer in healthy asymptomatic people: A systematic review and meta-analysis comparing risk ratio with risk difference

**DOI:** 10.1371/journal.pone.0183321

**Published:** 2017-08-17

**Authors:** Takeshi Seta, Yoshimitsu Takahashi, Yoshinori Noguchi, Satoru Shikata, Tatsuya Sakai, Kyoko Sakai, Yukitaka Yamashita, Takeo Nakayama

**Affiliations:** 1 Department of Gastroenterology and Hepatology, Japanese Red Cross Wakayama Medical Center, Wakayama, Japan; 2 Department of Health Informatics, Kyoto University School of Public Health, Kyoto, Japan; 3 Division of General Internal Medicine, Japanese Red Cross Nagoya Daini Hospital, Nagoya, Japan; 4 Department of Family Medicine, Mie Prefectural Ichishi Hospital, Tsu, Japan; 5 Department of Internal Medicine, Kyoto Katsura Hospital, Kyoto, Japan; 6 Department of Laboratory Medicine, Suita Saiseikai Hospital, Suita, Japan; National Cancer Center, JAPAN

## Abstract

**Background:**

*Helicobacter pylori* infection is strongly associated with gastric cancer occurrence. However, it is unclear whether eradication therapy reduces the risk of gastric cancer occurrence. We evaluated whether *H*. *pylori* eradication reduces the risk of primary gastric cancer by using both risk ratio (RR) and risk difference (RD).

**Methods:**

Searches of PubMed, EMBASE, Google scholar, the Cochrane Library, and the Japan Medical Abstracts Society as well as those registered in databases of the Cochrane Central Register of Controlled Trials, metaRegister of Controlled Trials, ClinicalTrials.gov, controlled-trials.com, UMIN-CTR, JMACCT-CTR, and JAPIC-CTI between January 1965 and March 2017, supplemented with manual screening. Randomized controlled trials (RCTs) in which eradication therapy were implemented for the interventional group but not for the control group, and assessed the subsequent occurrence of primary gastric cancer as the main outcome. Two authors independently reviewed articles and extracted data. Integrated results for all data were presented as RR and RD.

**Results:**

Seven studies met inclusion criteria. The reductions in risk of primary gastric cancer occurrence in terms of overall RR and RD were 0.67 (95% CI: 0.48 to 0.95) and -0.00 ([95% CI: -0.01 to 0.00]; number needed to treat: 125.5 [95% CI: 70.0 to 800.9]), respectively.

**Conclusions:**

The effectiveness of *H*. *pylori* eradication therapy in suppressing the occurrence of primary gastric cancer was significant and comparable to that of previous studies in terms of the estimated RR. However, the estimated RD was slight and not statistically significant.

## Introduction

*Helicobacter pylori* was designated a definite carcinogen of gastric cancer in 1994 by the International Agency for Research on Cancer (IARC) [1]. Infection of the gastric mucosa eventually leads to cancer via superficial gastritis, atrophic gastritis, intestinal metaplasia, and dysplasia [2]. Take *et al*. confirmed reductions in gastric cancer occurrence in follow-up studies of patients with peptic ulcers who had received eradication therapy [3, 4]. Fukase *et al*. showed in a randomized controlled trial (RCT) that *H*. *pylori* eradication therapy following endoscopic treatment of early-stage gastric cancer reduced the occurrence of metachronous gastric cancer by approximately 30% [5].

Fuccio *et al*. conducted a meta-analysis of seven RCTs that had been published in 2009, and reported that *H*. *pylori* eradication significantly reduces primary gastric cancer by roughly 35% in patients with gastritis or precancerous lesions [6]. However, this analysis included an RCT, in which endoscopic resection of early-stage gastric cancer suppressed the occurrence of metachronous cancer [5], as well as a set of multiple articles for a single study [7]. Ford and Moayyedi re-examined these data when these studies were excluded, and found that although the effect size was comparable, the reduction was no longer significant [8]. Subsequently, Ford *et al*. conducted systematic reviews within Asia of gastritis or precancerous lesions triggered by *H*. *pylori* and demonstrated that gastric cancer occurrence may be prevented by eradication therapy, based on the results of six RCTs published in 2013 [9, 10]. In 2016, Lee, *et al*. updated the systematic review and showed a similar result [11].

Risk ratio (RR), a synonym for relative risk, means the ratio of two risks, usually of exposed and not exposed [12]. An RR of <1 means the event is less likely to occur in the interventional group than in the control group. Risk difference (RD), a synonym for attributable risk, means the difference of two risk, usually risk in the exposed minus risk in the unexposed. When the probability of primary endpoint of intervention group and placebo group is low, it is sometimes replaced by a large number in terms of risk ratio despite being slightly expressed by risk difference. These four systematic reviews [6, 9–11] only used the risk ratio (RR) as an integrative index. In risk communication, the ratio index is concerned to exaggerate the effect size compared to the absolute value or difference index [13, 14]. In based on the absolute value is inevitable for evaluating the size of the problem and is also useful for the deployment of policies. Although it has been repeatedly and strongly recommended to combine the risk difference and the risk ratio [15–19], it has been reported that the risk difference compared to the risk ratio is reported less [20–22]. Especially in the issue of primary prevention, in general, the absolute risk is small and the effect size of the intervention shown with the risk ratio will give an exaggerated impression.

This study aimed to re-examine the significance of *H*. *pylori* eradication therapy in suppressing gastric cancer by expanding the period and range of the literature search and by showing effect sizes with RR and risk difference (RD).

## Methods

This systematic review and meta-analysis followed the PRISMA guidelines [23].

### Literature search

We searched the literature for RCTs published between January 1965 and March 2017, using PubMed, EMBASE, Google scholar, and the Cochrane library as English literature databases. Considering the high prevalence of gastric cancer in Japan and the availability of Japanese research articles, we also searched the Japan Medical Abstracts Society database [24]. Search terms were as follows: *Helicobacter pylori*, gastric cancer, intestinal metaplasia, gastric atrophy, dysplasia, atrophic gastritis, and chronic gastritis (final search conducted in April 2017). Electronic search strategy for PubMed database was shown in Table 1.

**Table 1:** Electronic search strategy for PubMed database.

Search (((((randomized controlled trial) OR (randomized controlled trials))) AND ((chronic gastritis) OR (gastritis) OR (atrophic gastritis) OR (dysplasia) OR (gastric atrophy) OR (gastric metaplasia) OR (intestinal metaplasia))) AND helicobacter pylori) AND gastric cancer Filters: Humans; English; Japanese

A search was also conducted for clinical trials that had been registered by March 2017, using the following databases: the Cochrane Central Register of Controlled Trials (CENTRAL) [25], metaRegister of Controlled Trials (mRCT) [26], ClinicalTrials.gov [27], controlled-trials.com [28], University Hospital Medical Information Network in Japan Clinical Trials Registry (UMIN-CTR) [29], Center for Clinical Trials, Japan Medical Association Clinical Trial Registry (JMACCT-CTR) [30], and Japan Pharmaceutical Information Center Clinical Trials Information (JAPIC-CTI) [31] (final search conducted in April 2017). In addition, we searched for previous systematic reviews and meta-analyses on the same topic. After this, we read through all titles and abstracts of the following representative Japanese journals in gastroenterology: *“I to Cho”* (Stomach and Intestine, published by Igaku-Shoin Ltd., Tokyo), *“Shokakinaishikyou”* (Endoscopia Digestiva, published by Tokyo Igakusha Ltd., Tokyo), and *“Rinsho Shokakinaika”* (Clinical Gastroenterology, published by Nihon Medical Center Inc., Tokyo) (final search conducted in April 2017). We then read through the entire text of articles related to the present topic. In cases where the same study participants were observed for gastric cancer occurrence at different times, we used the research article with the longest follow-up period.

Finally, we checked for studies in which multiple articles resulted from a single study, wherein extended observations of the same research participants included in the initial phase of the study were reported after the observations from the initial study had been published. Specifically, we contacted corresponding authors via e-mail and asked whether a single study resulted in multiple articles [32]; we decided in advance that if there was no reply after one week, a reminder e-mail would be sent to the author, and that if there was still no reply, the follow-up would be discontinued.

We used the “PRISMA-2009-Checklist” to evaluate the quality of literature search and eligibility criteria for this systematic review and meta analysis in S1 Table.

### Eligibility criteria

#### Inclusion criteria

Inclusion criteria were as follows: an RCT of *H*. *pylori* eradication therapy, or a study in which the occurrence of primary gastric cancer was subsequently followed up; an interventional group involving eradication therapy; a control group given “a placebo,” “no treatment (observation),” “a supplement,” or “an antacid containing a proton pump inhibitor (PPI),” none of which eradicated *H*. *pylori*; published in either English or Japanese; evidence of *H*. *pylori* infection demonstrated by a biochemical, serological, bacteriological, or histological method; and absence of gastric cancer as determined in advance by upper gastrointestinal endoscopy. Inclusion did not depend on the presence or absence of symptoms during participation in the study.

#### Exclusion criteria

Exclusion criteria were as follows: administration of eradication therapy in the control group; eradication therapy prior to group allocation; studies in which gastric cancer occurrence was not measured; studies that tracked whether metachronous cancer occurred after endoscopic therapy (endoscopic mucosal resection (EMR) or endoscopic submucosal dissection (ESD)) for primary cancer; animal studies; basic medical research; pathological research; reviews; and guidelines.

### Main outcome measure

We designated primary gastric cancer occurrence as the outcome, regardless of whether the measure was the primary or secondary outcome in each study.

### Data extraction

Based on eligibility criteria, two authors (T.S. and T.N.) independently checked the titles and summaries of all articles, searched and determined which were appropriate for inclusion. The other co-authors checked every process, and any issues that arose were resolved by discussion. The entire text of included articles was read, and the following information was extracted: year of publication; publication format; protocol; number of participating facilities in each study; subject characteristics; study country; language of publication; presence or absence of upper gastrointestinal symptoms at study initiation; method of eradication therapy; type of treatment in the control group; whether or not secondary eradication therapy was performed; treatment period; method of assessing *H*. *pylori* infection; number of times successful eradication was confirmed; primary and secondary outcomes; description of changes in gastric mucosa following eradication therapy; mean age; whether or not a difference was observed between the interventional and control groups; number of subjects; gastric cancer occurrence; histological type of gastric cancer; site of gastric cancer; degree of gastric cancer progression; gastric histological features at study initiation; histological assessment using updated Sydney System scores before and after eradication therapy [33]; proportion of subjects showing either intestinal metaplasia or dysplasia; clearly defined rationale for specifying the follow-up period; follow-up period; *H*. *pylori* eradication rate; drop-out rate; mean time to cancer occurrence; and time between endoscopies. Atrophic gastritis, intestinal metaplasia, and dysplasia were defined as precancerous lesions. We also noted whether there were multiple articles for a single study. If differences arose regarding details of the extracted data, all authors continued discussions until a consensus was achieved.

### Quality assessment for primary trials

The quality of primary trials was assessed as described by Jadad *et al* [34]. This method assesses whether the trial is randomized, the appropriateness of randomization if present, whether the trial is double-blinded, the appropriateness of double-blinding if present, and withdrawals/dropouts, using a score of 0 or 1 for each item. Total scores thus range from 0 to 5. A high quality trial in this meta-analysis was defined by a Jadad score of ≥3 points. The GRADE system was used to evaluate the risk of bias of each trial used in this meta-analysis [35]. An RCT was considered high quality if three or more of the six domains for assessing risk of bias were adequate. Ultimately, the quality of a study was determined by either the Jadad score or risk of bias.

### Statistical analysis

In four previous meta-analyses [6, 9–11], the pooled RR was used as the main index. Because this index tends to overestimate the effect, we first used the pooled RD and then the pooled RR, after which the results were presented together. The RD was calculated as risk in the interventional group *minus* that in the control group. The RD described the absolute change in risk that was attributable to the intervention. In other words, if the intervention had an identical effect to the control, RD would be 0. If it reduced the risk, the risk difference would be less than 0; if it increased the risk, the RD would be bigger than 0. The RD ranged from -1 to 1. A “+” sign indicated that treatment was favored, while a “-” sign indicated that the control was favored. Afterwards, the weighted pooled estimates were calculated for binary data. A fixed-effect model weighted by the Mantel-Haenszel (M-H) method was used to pool RD [36], followed by a test of homogeneity. Homogeneity among trials was assessed using the I^2^ test [37]. If the hypothesis of homogeneity was rejected, a random-effect model using the DerSimonian-Laird method was employed [38]. The potential for publication bias was examined by the funnel plot method [39], and the statistical significance of differences was evaluated in accordance with the methods of Begg or Egger [40, 41]. Given the observed risk difference, the number of patients that need to be treated (NNT) to prevent one adverse effect was also used as a measure of treatment effect; computationally speaking, NNT = 1/RD. Furthermore, the impact of eradication therapy, compared with placebo or no treatment, was expressed as a relative risk of occurrence of gastric cancer with 95% confidence intervals (CIs). All statistical analyses were performed with STATA statistical software version 14 [42]. Results are expressed as means and 95% CIs, unless indicated otherwise. P values < 0.05 were considered statistically significant. In addition, in order to evaluate visually whether the suppressive effect of eradication therapy on gastric cancer tends to change with time, we plotted the RD on the y-axis against the mean follow-up period for the interventional and control groups of each study on the x-axis, and fit a simple linear regression line based on the least squares method.

## Results

### Search results

**Fig 1:**
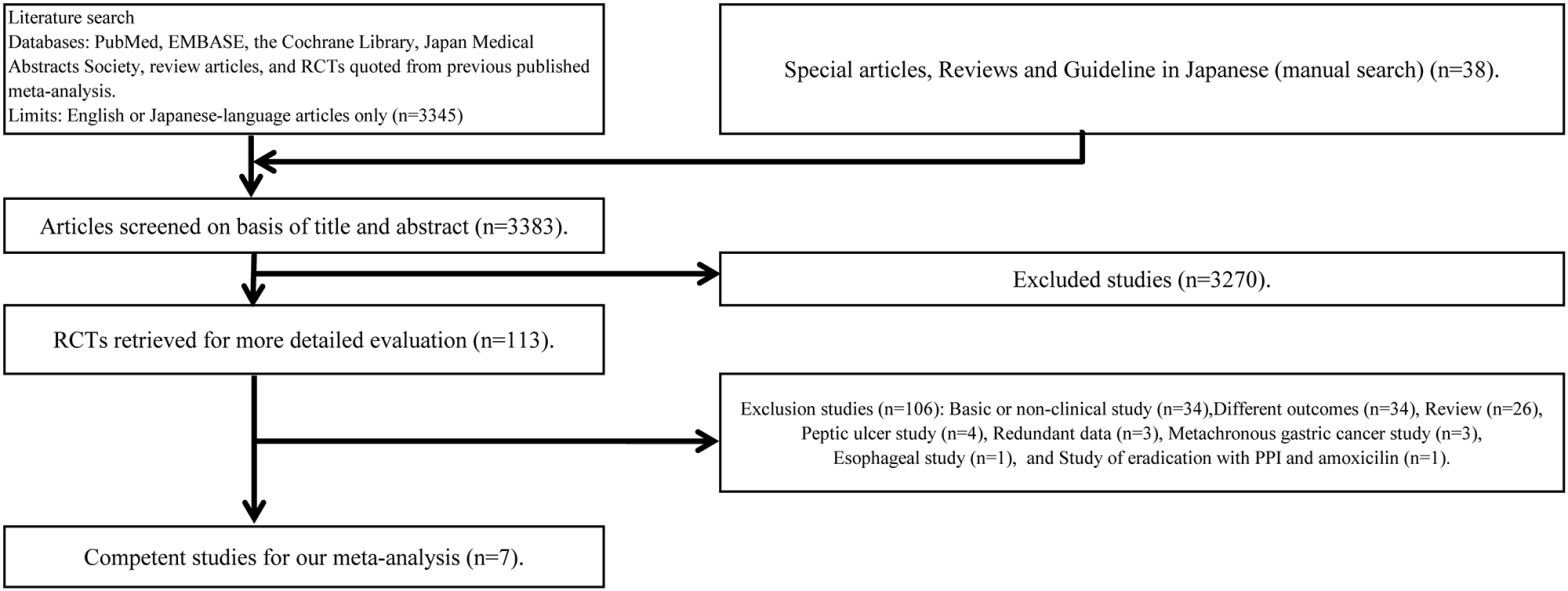
Flow of randomized controlled trials through the process of retrieval and inclusion in the meta-analysis comparing eradication treatment for Helicobacter pylori infection. RCT, Randomized controlled trial.

We screened 3383 studies through database searches and by reading through publications. Of these, we excluded 3270 studies and evaluated 113 in detail, and were ultimately left with seven studies (Fig 1) [43–49]. An RCT by Wong *et al*. compared four groups including a placebo group [48]. Of these, the two groups that met the inclusion criteria and were thus extracted for this study included one for which treatment intervention was a typical eradication therapy, and placebo administration for the other. Of the 106 studies excluded, we confirmed three article sets that involved multiple articles for a single study, in which the subjects and study background were identical but the study period differed. The studies by Zhou *et al*.[49], Mera *et al*.[45], and Ma *et al*.[47] were the long-term versions of those by Leung *et al*.[7], Correa *et al*.[50], and You *et al*. [51], respectively. Prior to their final report [49], Zhou *et al*. had also published a summarized version [52]. In accordance with the methodology described above, we excluded short-term studies with the same background and ultimately included their long-term counterparts (each published after the short-term study). Although the study by Miehke *et al*.[44] was not included in previous four meta-analyses [6, 9–11], we included it here as it met our inclusion criteria. None of the RCTs in the Japan Medical Abstracts Society or the three gastrointestinal medical journals satisfied the inclusion criteria. The article by Saito *et al*. was an abstract published as a poster at an academic conference (Digestive Disease Week, DDW 2005) [46]. Because the subsequent clinical study was not presented as an article, only the conference abstract was used. The research results were first published in Japanese; we did not find any article that later reported the same results in English.

### Study characteristics

Characteristics of all seven studies included in the final analysis [43–49] are summarized in Table 2. An intention-to-treat (ITT) analysis had been carried out in all of the studies, which were all published in English. Five of the seven studies were multicenter studies [43, 44, 46, 47, 49]. Subjects were normal healthy individuals in four studies [44–46, 48] and unspecified in three [43, 47, 49]; none of the studies clearly stated that the subjects were patients. Upper abdominal symptoms during the study were absent in three studies [44–46] and unspecified in four [43, 47–49]. The countries participating in the studies were China in four studies [44, 47–49]; Japan in one [46]; Columbia in one [45], and Austria, Czech Republic, and Germany in a jointly reported study [43]. Treatment details were as follows:

**Table 2:** Characteristics of primary trials.

Author	Reference	Year	Format	Setting	Participants' background		Country	Treatment of			Method of assessing *Helicobacter pylori* infection	Outcome		Mean age of		Participants in the initial allocation	
					Hospital	Participants		Intervention group	Control group	Duration (days)		Primary	Secondary	Intervention group	Control group	Intervention group	Control group
Miehlke S	43	2001	Full	Multicenters	University and general hospitals	Unclear	Austria, Czech Republic, and Germany	Omeprazole, 20mg; clarithromycin, 500mg; and amoxicillin, 1000mg, all twice daily	PPI	7	Histology ^13^C-urea breath test	Incidence of gastric cancer	None	Unclear	Unclear	86	81
Wong BCY	44	2004	Full	Multicenters	Public health bureau	Good health	China	Omeprazole, 20mg; metronidazole, 400mg; and amoxicillin, 750mg, all twice daily	Placebo	14	^13^C-urea breath test	Incidence of gastric cancer	None	42.0	42.0	817	813
Mera R	45	2005	Full	Single	Public health bureau	Good health	Colombia	Amoxicillin; metronidazole; and bismuth subsalicylate	Supplement	14	Histology	Mucosal healing	Incidence of gastric cancer	Unclear	Unclear	394	401
Saito D	46	2005	Abstract	Multicenters	University and general hospitals	Good health	Japan	Lansoprazole, 30mg; clarithromycin, 200mg; and amoxicillin, 750mg, all twice daily	None	7	Unclear	Mucosal healing	Incidence of gastric cancer	Unclear	Unclear	379	313
Ma JL	47	2012	Full	Multicenters	Unclear	Unclear	China	Lansoprazole, 30mg; clarithromycin, 200mg; and amoxicillin, 750mg, all twice daily	Placebo	7	^13^C-urea breath test Serum antibody	Incidence of gastric cancer	None	47.0	47.0	1130	1128
Wong BCY	48	2012	Full	Multicenters	University	Unclear	China	Omeprazole, 20mg; clarithromycin, 500mg; and amoxicillin, 1000mg, all twice daily	Placebo	7	^13^C-urea breath test	Mucosal healing	Incidence of gastric cancer	53.0	52.9	255	258
Zhou LY	49	2014	Full	Single	Public health bureau	Good health	China	Omeprazole, 20mg; clarithromycin, 500mg; and amoxicillin, 1000mg, all twice daily	Placebo	7	^13^C-urea breath test	Mucosal healing	Incidence of gastric cancer	62.1	62.2	276	276

Patients with gastric cancer										Follow up (year)		Patients lost to follow-up (%)		Method of the contact to the correspondance author	Existence of the reply to the question to the correspondance author	Contents of the reply		Reaction of the remind mail
Intervention group					Control group													
Overall	Intestine type	Gastric type	Cardic cancer	Non-cardic cancer	Overall	Intestine type	Gastric type	Cardic cancer	Non-cardic cancer	Intervention group	Control group	Intervention group	Control group			Redundant data	Unpublicated data	
0	0	0	0	0	0	0	0	0	0	1.3	1.3	Unclear	Unclear	E-mail	No	Unclear	Unclear	No
7	Unclear	Unclear	Unclear	Unclear	11	Unclear	Unclear	Unclear	Unclear	7.5	7.5	10	14	E-mail	No	Unclear	Unclear	No
5	Unclear	Unclear	Unclear	Unclear	4	Unclear	Unclear	Unclear	Unclear	12.0	12.0	Unclear	Unclear	E-mail	Connection was impossible	Unclear	Unclear	-
2	Unclear	Unclear	Unclear	Unclear	3	Unclear	Unclear	Unclear	Unclear	≧4	≧4	Unclear	Unclear	Adress was unknown	-	Unclear	Unclear	-
34	Unclear	Unclear	Unclear	Unclear	52	Unclear	Unclear	Unclear	Unclear	14.7	7.3	6	8	E-mail	No	Unclear	Unclear	No
3	3	0	Unclear	Unclear	1	0	1	Unclear	Unclear	5.0	5.0	8.6	9.3	E-mail	No	Unclear	Unclear	No
2	Unclear	Unclear	Unclear	Unclear	7	Unclear	Unclear	Unclear	Unclear	10.0	10.0	33	30	E-mail	Connection was impossible	Unclear	Unclear	-

PPI, Proton pump inhibitor

Treatment in the intervention group was PCA (PPI + clarithromycin + amoxicillin) in five studies [43, 46–49], BMA (bismuth + metronidazole + amoxicillin) in one [45], and PMA (PPI + metronidazole + amoxicillin) in one [44]. Treatment in the control group was placebo in four studies [44, 47–49], a supplement in one [45], no treatment in one [46], and PPI in one [43]. With the exception of one study [45], none of the studies had performed secondary *H*. *pylori* eradication therapy. The method for assessing *H*. *pylori* infection was the ^13^C-Urea breath test in five studies [43, 44, 47–49], histology in two [43, 45], serum antibodies in one [49], and unspecified in one [47]. The number of times the effectiveness of *H*. *pylori* eradication was confirmed was only once in five studies [43–45, 47, 49], four times in one (by a breath test at the time of each endoscopy) [48], and unspecified in one [46].

In terms of outcome evaluation, gastric cancer occurrence had been designated as the primary outcome measure in three studies [43, 44, 49], while a secondary outcome measure was specified in four [45–48]. In an upper gastrointestinal endoscopic examination following eradication therapy, one study [45] found significant improvement of the gastric mucosa whereas three found no significant improvement [46–48]; the other three did not assess this parameter [43, 44, 49].

The total numbers of subjects for the interventional and control groups were 3337 and 3270, respectively, with a mean age of 51.0 years for both groups. Mean follow-up periods for the interventional and control groups were 7.8 and 6.7 years, respectively; the rationale for the follow-up period was not indicated in any study. Two studies [44, 48] reported the time to cancer onset following eradication therapy, and indicated mean times of 3.9 and 2.3 years for the interventional and control groups, respectively.

Histological assessment, using the updated Sydney System score before and after eradication therapy, was not possible for any of the studies. Endoscopy was performed annually in one study [43] or following a fixed non-annual schedule in four studies [44, 45, 47, 48]; the schedule was unspecified in two studies [46, 49].

We sent e-mail inquiries to six of the eight corresponding authors [43–45, 47–49] whose addresses were listed, asking if multiple articles with redundant data had been published. E-mails sent to the listed addresses for two of these six authors did not reach their destinations [45, 49]. None of the remaining four authors replied within the first week; therefore, we sent a reminder to all of these authors [43, 44, 47, 49]. Because we received no replies even after another week, we conducted no further follow-ups.

### Study quality

We first evaluated the quality of the seven RCTs in Table 3. The median Jadad score was 3.0 (range: 1–5). The primary outcome measure was described in the methodology of every study. Sample size had been specified in advance in three studies [44, 47, 49]. The funding source was public in four studies [44, 45, 47, 49] and unspecified in three [43, 46, 48]. None of the studies mentioned whether there were any conflicts of interest. In addition, although one study indicated that drugs were actually provided, six lacked such description [43–48].

**Table 3:** Evidence quality of each RCT used.

Author	Reference	Description of			Description of grant		Limitations (Risk of bias) for each RCT						Jadad score						Quality of study
		Main outcome in methods	Basis of sample size calculation in methods	Fund	Fund was supplied by sponsor company	Medicine was supplied by sponsor company	Allocation concealment	Adequate sequence generation	Blinding	Incomplete outcome data addressed	Free of elective outcome reporting	Free of other bias	Randomization	Appropriateness of randomization	Double blind	Appropriateness of double blind	Dropout	Sum	
Miehlke S	43	Yes	No	No	No	No	Yes	Yes	Yes	Yes	Yes	Unclear	1	0	1	0	1	3	High
Wong BCY	44	Yes	Yes	Public	No	No	Yes	Yes	Yes	Yes	Yes	Unclear	1	1	1	1	1	5	High
Mera R	45	Yes	No	Public	No	No	No	Yes	No	Unclear	Yes	Unclear	1	0	0	0	1	2	Low
Saito D	46	Yes	No	No	No	No	No	Yes	No	Unclear	Yes	Unclear	1	0	0	0	0	1	Low
Ma JL	47	Yes	Yes	Public	No	Yes	No	Yes	No	Unclear	Yes	Unclear	1	0	1	0	0	3	High
Wong BCY	48	Yes	Yes	Public	No	No	Yes	Yes	Yes	Unclear	Yes	Unclear	1	1	1	0	1	4	High
Zhou LY	49	Yes	No	No	No	No	No	Yes	No	Unclear	Yes	Unclear	1	0	1	0	1	3	High

RCT, Randomized controlled trial

Regarding the risk of bias for each RCT, three studies mentioned *allocation concealment* [43, 44, 47], all noted *adequate sequence generation*, and three mentioned *blinding* [43, 44, 49]. Only two studies noted *inadequate outcome data* [43, 44], and *free of elective outcome reporting* was mentioned in all studies. None of the studies commented on whether they were *free of other biases*. Consequently, the quality of each study was moderate, as was the quality of the body of evidence in general.

### Suppressive effect of *H*. *pylori* eradication therapy on primary gastric cancer

**Fig 2:**
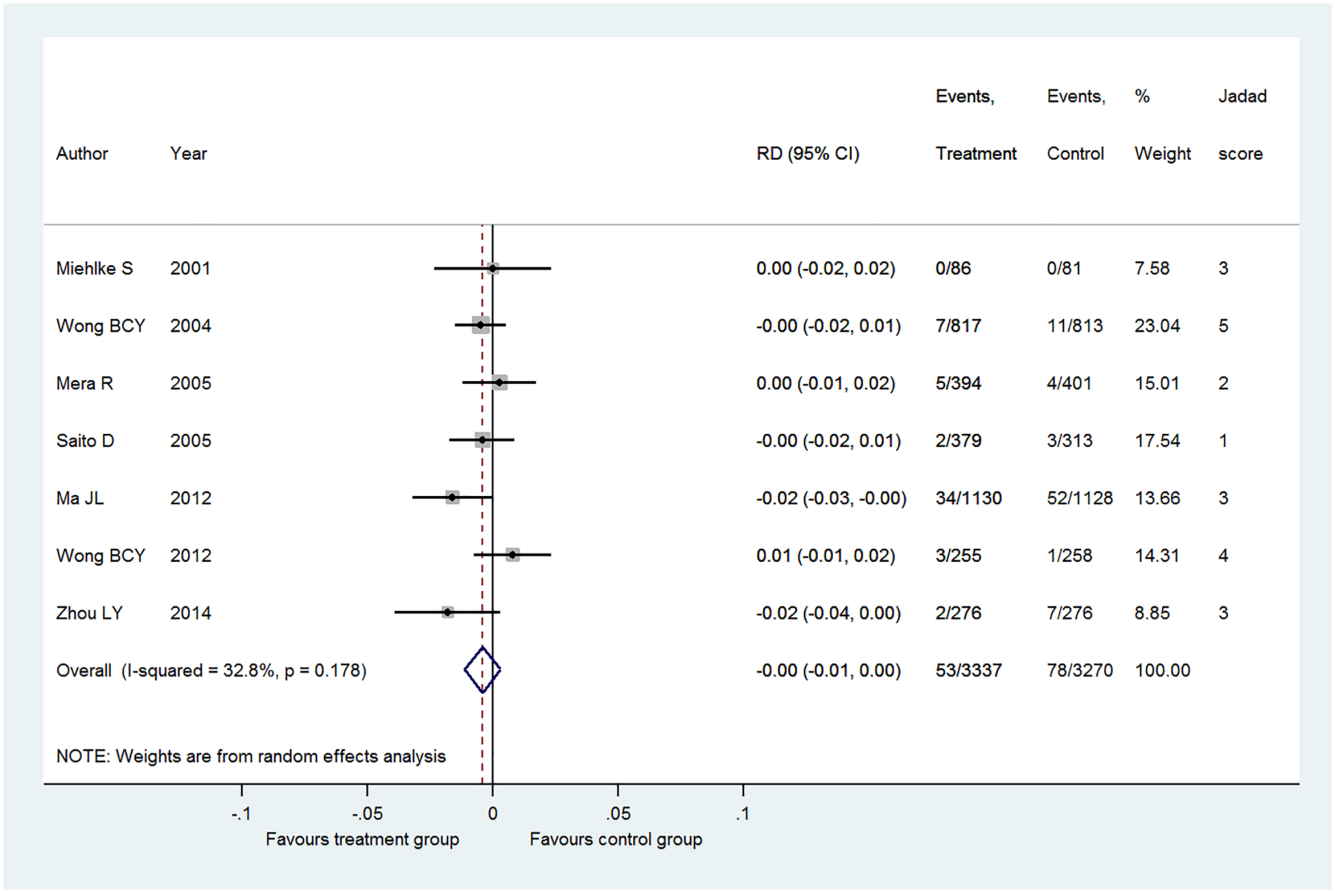
Pooled risk difference (RD) in gastric cancer occurrence in patients with *Helicobacter pylori* infection. I^2^ value indicates heterogeneity of 33%. n = case of gastric cancer. N = group size.

Overall, eradication therapy of *H*. *pylori* infection significantly reduced the risk on primary gastric cancer (pooled risk ratio [RR], 0.67; 95% Confidence Interval [CI], 0.48 to 0.95 with low heterogeneity (I^2^ = 0%) in Table 4 and Fig 2. A subgroup analysis, the pooled RR was significantly shown for RCTs of high quality, those within Asia, those in which the control group was given a placebo or no treatment, those that targeted subjects with a mean age between 41 and 50 years, and even those in which the mean study period exceeded 10 years.

**Table 4:** Effectiveness of Helicobacter pylori eradication therapy for gastric cancer prevention.

Outcomes	Reference	No. of studies	Pooled risk ratio			Heterogeneity	Pooled risk difference			Heterogeneity	Statistical method by effect model	NNT			Quality of a body of evidence
			Value	95%CI		I^2^ value (%)	Value	95%CI		I^2^ value (%)		Value	95%CI		
				Lower	Upper			Lower	Upper				Lower	Upper	
Overall	43–49	7	0.67	0.48	0.95	0	-0.00	-0.01	0.00	33	D-L	125.5	70.0	800.9	Moderate
High quality RCTs	43, 44, 47–49	5	0.65	0.45	0.93	0	-0.01	-0.02	0.00	50	D-L	101.7	57.4	604.0	
RCT in Japan	46	1	0.55	0.09	3.27	Uncalculatable	-0.00	-0.02	0.01	Uncalculatable	-	232.1	79.8	∞	
Studies within Asia	44, 46–49	5	0.64	0.45	0.92	0	-0.01	-0.01	0.00	47	D-L	102.7	59.6	463.2	
Studies outside of Asia	43, 45	2	1.27	0.34	4.70	Uncalculatable	0.00	-0.01	0.02	0	M-H	472.2	85.7	∞	
Annual healthy screening or good health	44–46, 49	4	0.63	0.34	1.17	0	-0.01	-0.01	0.00	0	M-H	185.8	88.4	∞	
Giving placebo or no treatment to comparison group	44, 46–49	5	0.64	0.45	0.92	0	-0.01	-0.01	0.00	47	D-L	102.7	59.6	463.2	
Cases of gastric mucosal improvement after eradication	45	1	1.27	0.34	4.70	Uncalculatable	0.00	-0.01	0.02	Uncalculatable	-	368.3	70.1	∞	
Patients with preneoplastic lesions	44, 47, 48	3	0.69	0.47	1.00	0	-0.00	-0.02	0.01	68	D-L	77.7	38.0	∞	
Patients without preneoplastic lesions	43, 45, 46, 49	4	0.62	0.27	1.43	6	-0.00	-0.01	0.00	0	M-H	155.4	90.1	∞	
Participants' mean age, 41 to 50	44, 47	2	0.65	0.44	0.96	0	-0.01	-0.02	0.00	46	D-L	87.7	48.1	794.6	
Age 51 to 60	48	1	3.04	0.32	29.0	Uncalculatable	0.01	-0.01	0.02	Uncalculatable	-	126.8	70.1	∞	
Gastric cancer, gastric type	43, 48	2	3.04	0.32	29.0	Uncalculatable	0.01	-0.01	0.02	0	M-H	535.0	Uncalculatable		
Gastric cancer, intestinal type	43, 48	2	3.04	0.32	29.0	Uncalculatable	0.01	-0.01	0.02	0	M-H	535.0	Uncalculatable		
Gastric cancer, cardiac type	43	1	Uncalculatable			Uncalculatable	0.00	-0.02	0.02	Uncalculatable	-	Uncalculatable			
Gastric cancer, non cardiac type	43	1	Uncalculatable			Uncalculatable	0.00	-0.02	0.02	Uncalculatable	-	Uncalculatable			
Annual follow-up of endoscopic examination	43	1	Uncalculatable			Uncalculatable	0.00	-0.02	0.02	Uncalculatable	-	Uncalculatable			
Scheduled follow-up except annual endoscopic examination	44, 45, 48, 49	4	0.74	0.40	1.38	19	-0.00	-0.01	0.01	37	D-L	294.2	102.2	∞	
Research duration (mean), shorter than 5 years	43, 46	2	0.55	0.09	3.27	Uncalculatable	-0.00	-0.01	0.01	0	M-H	301.8	100.7	∞	
5 to 10 years	44, 48	2	0.98	0.25	3.89	37	0.00	-0.01	0.01	48	D-L	533.0	105.7	∞	
Longer than 10 years	45, 47, 49	3	0.65	0.44	0.96	4	-0.01	-0.02	0.01	55	D-L	82.5	45.0	833.3	
Eradication therapy, PCA	43, 46–49	5	0.65	0.44	0.95	0	-0.01	-0.02	0.01	53	D-L	86.9	49.4	489.3	
PMA	44	1	0.63	0.25	1.63	Uncalculatable	-0.00	-0.02	0.01	Uncalculatable	-	201.5	76.1	∞	
BMA	45	1	1.27	0.34	4.70	Uncalculatable	0.00	-0.01	0.02	Uncalculatable	-	368.3	70.1	∞	

CI, confidence intervals; NNT, Number needed to treat; RCT, Randomized controlled trial; M-H, Mantel-Haenszel; D-L, DerSimonian-Laird

PCA, Proton pump inhibitor, clarithromycin, and amoxicillin; PMA, Proton pump inhibitor, metronidazole, and amoxicillin; BMA, bismuth, metronidazole, and amoxicillin

**Fig 3:**
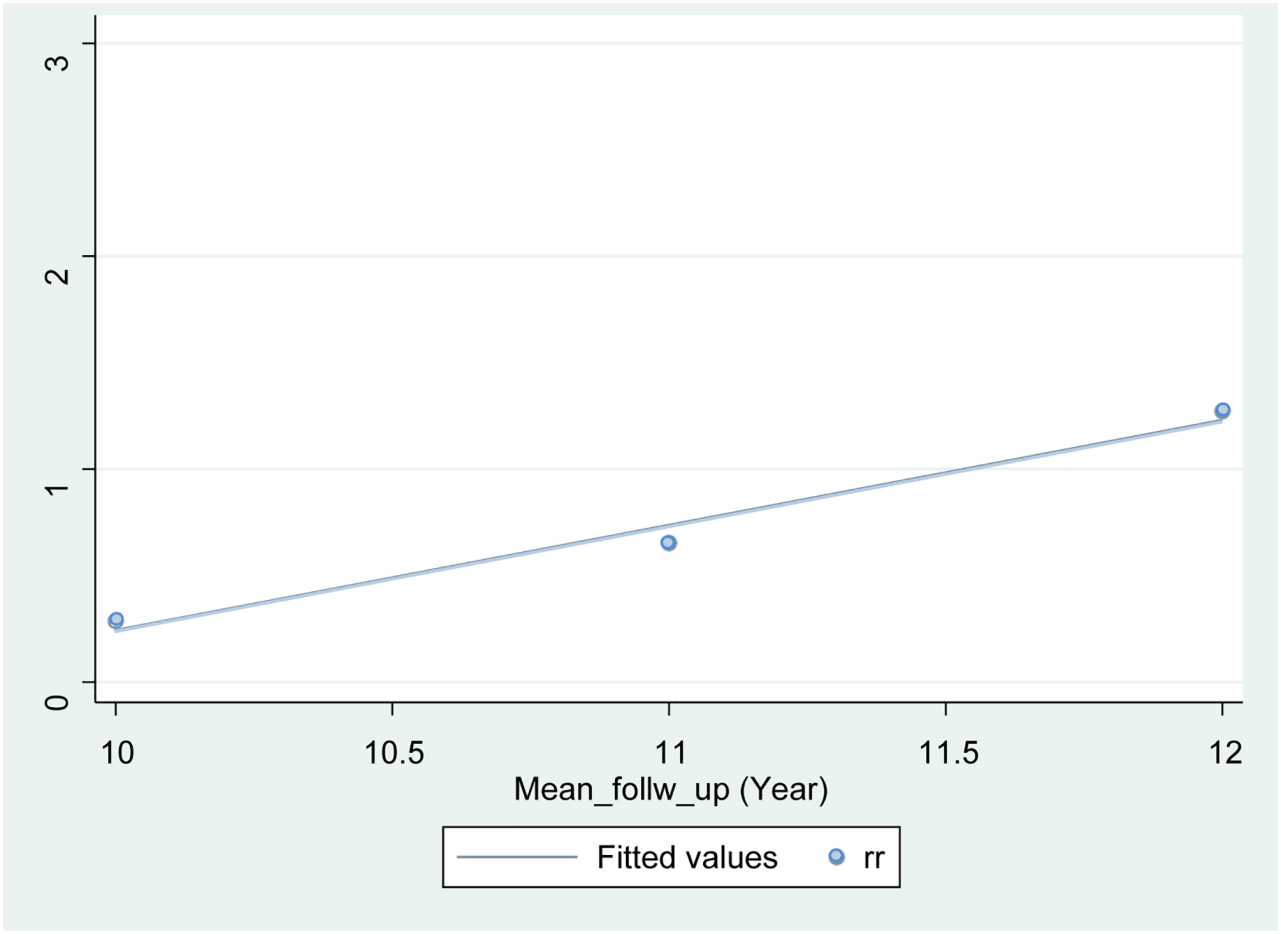
Simple linear regression. Risk ratio (y-axis) was plotted as a function of the mean follow-up period in the interventional and control groups of each study (x-axis), and a simple linear regression line was fitted using the least squares method. The point at which the risk of cancer occurrence in the interventional group exceeds that in the control group was calculated to be approximately 11.5 years. rr, risk ratio.

**Fig 4:**
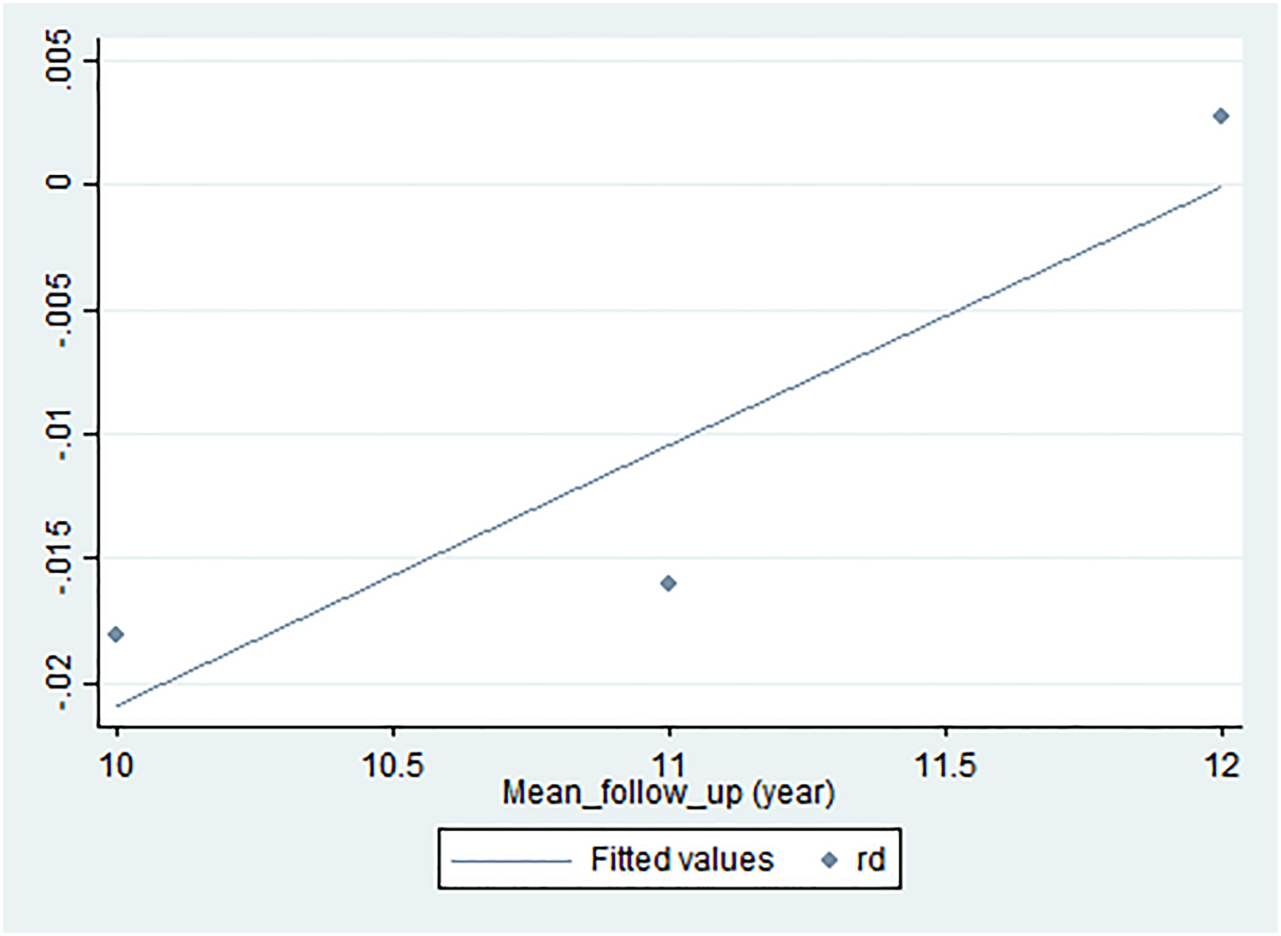
Simple linear regression. Risk difference (y-axis) was plotted as a function of the mean follow-up period in the interventional and control groups of each study (x-axis), and a simple linear regression line was fitted using the least squares method. The point at which the risk of cancer occurrence in the interventional group exceeds that in the control group was calculated to be approximately 12 years. rd, risk difference.

Overall, eradication therapy of *H*. *pylori* infection did not significantly reduce the risk on primary gastric cancer (pooled difference [RD], -0.00; 95% Confidence Interval [CI], -0.01 to 0.00 with low heterogeneity (I^2^ = 33%) and NNT = 125.5 [95% CI: 70.0 to 800.9]). When the analysis was restricted to the five high-quality RCTs, pooled RD was -0.01 [95% CI: -0.02 to 0.00] (I^2^ = 50%, NNT = 101.7 [95% CI: 57.4 to 604.0]). When analyzing the relationship between RR and follow-up period for the three studies with a mean follow-up period of at least 10 years [45, 48, 49], linear regression yielded a line with a positive slope (y (RR) = 0.4932x (year)– 4.6888) (Fig 3). Besides, when analyzing the relationship between RD and follow-up period for the three studies with a mean follow-up period of at least 10 years [45, 48, 49], linear regression yielded a line with a positive slope (y (RD) = 1.0416x (year)– 12.504) (Fig 4). This suggests that although the risk of cancer onset is higher in the control group than in the intervention group up to a follow-up period of 11.5 to 12 years, the relationship might be reversed beyond this point.

### Publication bias

**Fig 5:**
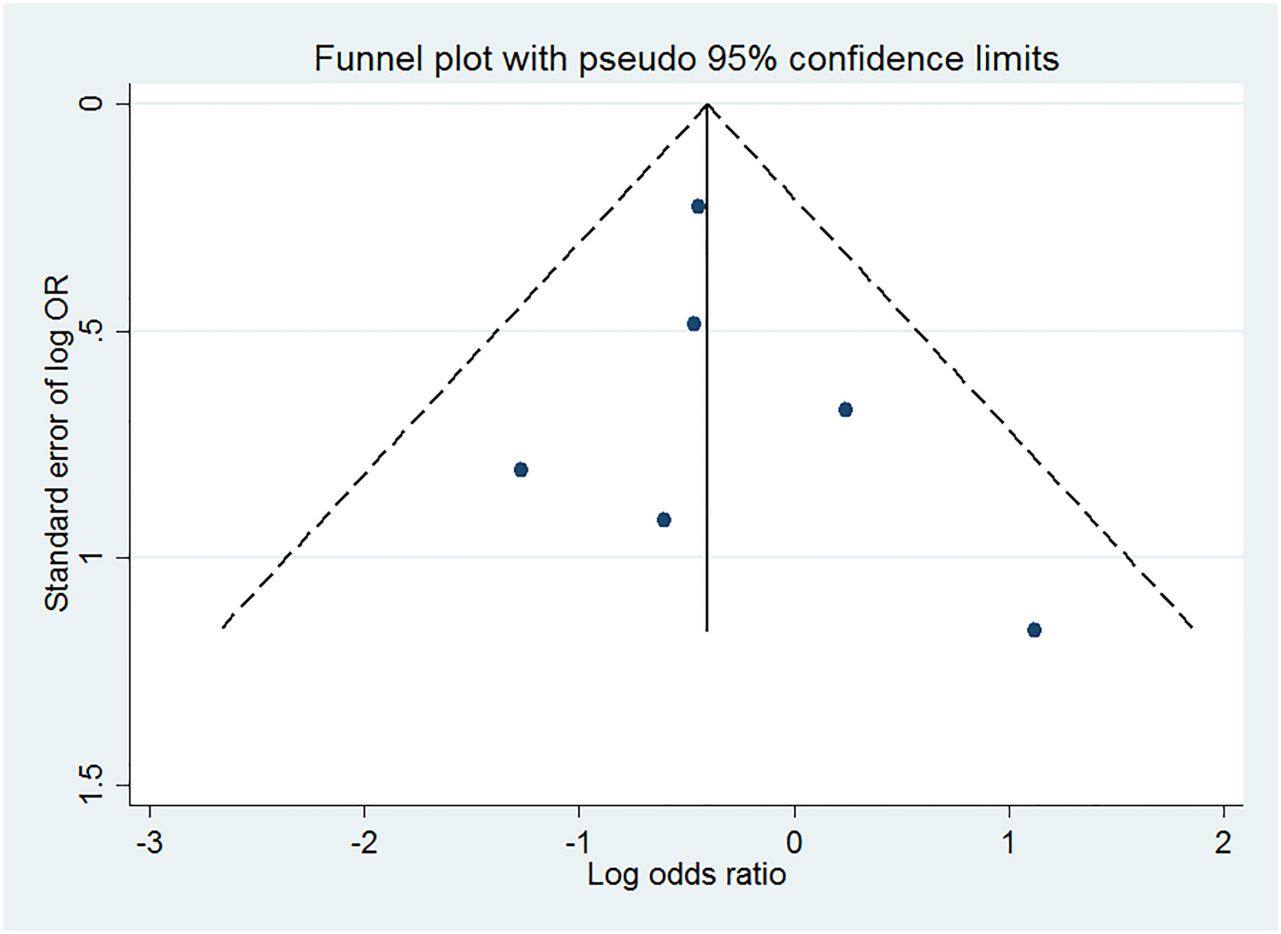
Publication bias of trials reporting gastric cancer in patients with *Helicobacter pylori* infection. OR, Odds Ratio.

Funnel-plot analysis, Begg’s test, and Egger’s test were performed to evaluate the potential for publication bias in terms of overall RD (Fig 5). The funnel-plot did not show an asymmetric pattern. Neither of the statistical tests revealed significant publication bias (p = 0.348, p = 0.610, respectively).

## Discussion

In this study, we conducted a systematic review of whether *H*. *pylori* eradication therapy suppresses the occurrence of primary gastric cancer in patients whose gastric cancer was not diagnosed endoscopically. Three RCTs [43, 45, 48] were added to the meta-analysis by Fuccio *et al*.[6] in 2009, and one [43] to the meta-analyses by Ford *et al*.[9, 10] in 2014 and 2015. A meta-analysis of the seven articles ultimately included revealed that, whereas the gastric cancer suppressing effect of eradication therapy was comparable to that of previous research in terms of RR, the effect size in terms of RD, which was not assessed in previous studies, was minor and not statistically significant. Overall NNT was 125, and subgroup analyses were approximately from 100 to 300. Showing these values, we think the effectiveness of eradication of *Helicobacter pylori* to prevent primary gastric cancer was relatively low. This trend was observed in all subgroup analyses in Table 4.

In this study, the overall pooled RR for the suppression of gastric cancer by *H*. *pylori* eradication was 0.67 (95% CI: 0.48 to 0.95), which was comparable to that of previous studies. However, when expressed in terms of RD, the overall pooled RD was -0.00 (95% CI: -0.01 to 0.00), i.e., the effect was slight and not statistically significant. It is common that "Relative Risk obtained from the same data looks larger than Risk Difference". Apart from whether it is one case symbolically seen in the present research question and whether it is deliberate or not intentional, in the previous study both RCT and meta-analysis were only indicators of risk ratio. The magnitude of the effect such as risk ratio 0.6–0.7 was emphasized, and the tone of the discussion was to justify the aggressive intervention. Although we used the same data to analyze pooled risk ratio and risk difference, pooled risk ratio was statistically significant but pooled risk difference was not. When the event-incidence was rare with meta-analysis, in another study, pooled risk ratio was statistically significant but pooled risk difference was not like our study [53]. We thought that the association between pooled risk ratio and risk difference showed discrepancy because we used data of rare events. In fact, Lane, *et al* showed that this discrepancy occurred when the methods of statistical tests in the meta-analysis were the differences and outcome incidence of the original data used in randomized controlled trials was rare. Main purpose of our study is to facilitate careful discussion on the expected magnitude of the effect of sterilization by *H*. *pylori* eradication to prevent primary gastric cancer comparing two risk indices that have been rarely mentioned before. We think that this phenomenon can be called a new outcome- reporting bias in risk communication. Many epidemiological findings, including those from clinical trials, are expressed in terms of RR; however, it has been repeatedly pointed out that compared with absolute risk, this index overestimates the association. [21]. For example, in a clinical trial by Lipid Research Clinics reported in 1982 [54], a 19% reduction in the risk of ischemic heart disease due to a cholesterol-reducing drug was emphasized. However, this was expressed in terms of relative risk reduction (RRR); the absolute risk reduction (ARR) was 1.6%. A similar report also emphasized an RRR of 31% over an ARR of 2.3% [55]. The RRR used in these studies, although not identical to relative risk, is a ratio-based index; this expression was likely used with the intent to strengthen reader impressions. Given these findings, the FDA proposed the following in a 2011 report when communicating risk: “Provide absolute risks, not just relative risks. Patients are unduly influenced when risk information is presented using a relative risk approach; this can result in suboptimal decisions” [13]. The RR results presented herein do not deny the effectiveness of *H*. *pylori* eradication in preventing cancer. Nonetheless, the results for absolute risk (AR) suggest that we should be cautious regarding the effect size and its level of certainty as evidence. Typically, the problem with selective reporting has been outcome reporting bias, in which numerous outcomes are measured and only those variables attaining statistical significance are published [56–58]. Previous studies of *H*. *pylori* eradication presented their results only as a ratio index that emphasizes its effect could be pointed out as a new selective reporting issue distinct from prevailing problems.

NNT is a treatment effect index based on ARR. Fuccio *et al*.[6] or Lee *et al*. [11] did not show NNT in their report. Although Ford *et al*.[9] showed NNT separately by country, their interpretation of this in their report was that the treatment would reduce the occurrence of gastric cancer within Asia. Obtaining the NNT for each population is appropriate because this index is influenced by the AR of a disease in a target population. However, a stable NNT cannot be obtained from findings in a single-population study that lacks power. To make evidence-based decisions, it will be necessary to alleviate the large impact that RR has on readers by obtaining NNT from an integrated RD and interpreting the results within the article.

To whom can we recommend *H*. *pylori* eradication therapy for suppressing the occurrence of gastric cancer? Eradication therapy is necessary for patients who have just undergone early-stage gastric cancer surgery [59–62] and is well-established for patients who have undergone endoscopic therapy for this type of cancer [5, 63]. This study, which targeted asymptomatic patients infected with *H*. *pylori*, predicted the following: first, that the reduction in the risk of gastric cancer by *H*. *pylori* eradication therapy would be at least 30% in terms of RR, similar to that of previous findings, but would be slight and statistically insignificant in terms of RD; and second, that after eradication, a suppressive effect on gastric cancer would likely occur in the short term but diminish in the long term due to increased occurrence of cancer from aging (Fig 3). Therefore, those with severe gastric mucosal atrophy or precancerous lesions, which are relatively elderly high-risk groups, tend not to benefit from the treatment effects. In contrast, young individuals without atrophy might be expected to benefit from the effects of eradication therapy in preventing gastric cancer. In the long-term, endoscopy will likely be necessary because cancer begins to occur more frequently with age, even in the intervention group.

This study has several limitations. First, the mean study periods of the RCTs used were short (6–7 years). In a review which cited an article by Graham *et al*. and the fact that the occurrence of cancer had also been demonstrated in RCTs with long-term follow-up, Tan *et al*. [64] stated that cohort studies on the same topic should also be referred to in evaluating the gastric cancer suppressing effects of *H*. *pylori* eradication therapy. Considering that many of the RCTs used here were completed within a few years, a re-examination including a cohort study with a longer observation period would be meaningful. For observational studies, pre-registration has not become as widespread as it has for RCTs; such studies should also be interpreted cautiously because publication bias is more severe than it is for interventional studies, and because cohort studies, given their high bias risk, may show results that diverge from true results. Second, the eradication effect could vary depending on histopathology, i.e., gastritis, atrophy, intestinal metaplasia, or dysplasia. However, these could not be clearly distinguished in the present study.

There is ABC classification for assessing gastric cancer risk and evaluating the degree of gastric mucosal atrophy objectively and quantitatively [65]. In our study, we couldn’t compare the degree of gastric mucosal atrophy quantitatively. Among youths, a *H*. *pylori* carrier without gastric mucosal atrophy could possibly lower the risk of primary gastric cancer [66]. This problem could be addressed by future studies with the development of a standardized method for tissue evaluation.

From the present study, we conclude that the suppressive effect of *H*. *pylori* eradication therapy on the occurrence of primary gastric cancer was statistically significant and comparable to that of previous studies in terms of estimated RR. However, in terms of RD, the effect size was minor and not statistically significant. At this point, caution must be exercised when promoting evidence-based eradication measures.

## Supporting information

S1 Table PRISMA-2009-Checklist. (DOC)

## Abbreviations

AR: Absolute risk
ARR: Absolute risk reduction
BMA: bismuth, metronidazole, and amoxicillin
CI: Confidence interval
COI: Conflict of interest
DDW: Digestive Disease Week
EMBASE: Excerpta Medica database
EMR: Endoscopic mucosal resection
ESD: Endoscopic submucosal dissection
FDA: Food and Drug Administration
ITT: Intention to treat
NNT: Number needed to treat
PCA: Proton pump inhibitor, clarithromycin, and amoxicillin
PMA: Proton pump inhibitor, metronidazole, and amoxicillin
PPI: Proton pump inhibitor
PRISMA: Preferred Reporting Items for Systematic Reviews and Meta-Analyses
RCT: Randomized controlled trial
RD: Risk difference
RR: Risk ratio
RRR: Relative risk reduction
